# The Relevance of the MCP Risk Polymorphism to the Outcome of aHUS Associated With C3 Mutations. A Case Report

**DOI:** 10.3389/fimmu.2020.01348

**Published:** 2020-07-16

**Authors:** Javier Lumbreras, Marta Subias, Natalia Espinosa, Juana María Ferrer, Emilia Arjona, Santiago Rodríguez de Córdoba

**Affiliations:** ^1^Unidad de Nefrología Infantil, Servicio de Pediatría, Hospital Universitari Son Espases–Instituto de Investigación Sanitaria Illes Balears (IdISBa), Palma de Mallorca, Spain; ^2^Centro de Investigaciones Biológicas Margarita Salas and Ciber de Enfermedades Raras, Madrid, Spain; ^3^Servicio de Inmunología, Hospital Universitari Son Espases–Instituto de Investigación Sanitaria Illes Balears (IdISBa), Palma de Mallorca, Spain

**Keywords:** C3, MCP risk polymorphism, atypical hemolytic uremic syndrome, *de novo* mutation, case report

## Abstract

Thrombotic microangiopathy (TMA) has different etiological causes, and not all of them are well understood. In atypical hemolytic uremic syndrome (aHUS), the TMA is caused by the complement dysregulation associated with pathogenic mutations in complement components and its regulators. Here, we describe a pediatric patient with aHUS in whom the relatively benign course of the disease confused the initial diagnosis. A previously healthy 8-year-old boy developed jaundice, hematuria, hemolytic anemia, thrombopenia, and mild acute kidney injury (AKI) in the context of a diarrhea without hypertension nor oliguria. Spontaneous and complete recovery was observed from the third day of admission. Persistent low C3 plasma levels after recovery raised the suspicion for aHUS, which prompted clinicians to discard the initial diagnosis of Shigatoxin-associated HUS (STEC-HUS). A thorough genetic and molecular study of the complement revealed the presence of an isolated novel pathogenic C3 mutation. The relatively benign clinical course of the disease as well as the finding of a *de novo* pathogenic C3 mutation are remarkable aspects of this case. The data are discussed to illustrate the benefits of identifying the TMA etiological factor and the relevant contribution of the MCP aHUS risk polymorphism to the disease severity.

## Introduction

Atypical hemolytic uremic syndrome (aHUS) is an ultra-rare disease characterized by acute kidney injury, thrombocytopenia, and microangiopathic hemolytic anemia, which results from an impaired protection of host endothelial cells from complement damage (1). The complement system is a key element of innate immunity with crucial roles in the elimination of pathogens, immune complexes, or cell remains. The complement activates by three pathways, classical (CP), lectin (LP), and alternative (AP), which generates protease complexes, named C3 convertases that cleave C3 to generate C3b. Convertase-generated C3b can form more AP C3 convertase, providing exponential amplification of the initial activation. Clustering of C3b around the surface-bound C3 convertase generates the C5 convertase, which cleaves C5 and initiates formation of the lytic membrane attack complex (MAC) (2). In health, the activation of C3 in plasma is kept at a very low level, and the deposition of C3b and further activation of complement are limited to the surface of pathogens by multiple regulatory proteins. The loss of complement regulation leads to the generation of proinflammatory components and/or tissue damage. Both situations have pathological consequences (3). Loss-of-function mutations in genes encoding the regulatory proteins factor H (FH), MCP, and factor I (FI), as well as gain-of-function mutations in the complement activating components factor B (FB) and C3, have been associated with aHUS (4–11). Criteria have been established to facilitate the clinical diagnosis of aHUS, but it is often difficult to exclude STEC-HUS and secondary HUS forms (12). Since 50–70% of aHUS patients have an underlying inherited and/or acquired complement abnormality (13, 14), genetic analyses are recommended to characterize the etiological factor, reinforce diagnosis, and assist patient management. We present a case that was initially classified as STEC-HUS but was reclassified to aHUS based on the complement follow-up and genetic analyses. We discuss the implications of the identification of a *de novo* gain-of-function C3 mutation in this case and the relevance of genotyping for the MCPggaac aHUS risk polymorphism.

### Clinical Case

**Figure 1:**
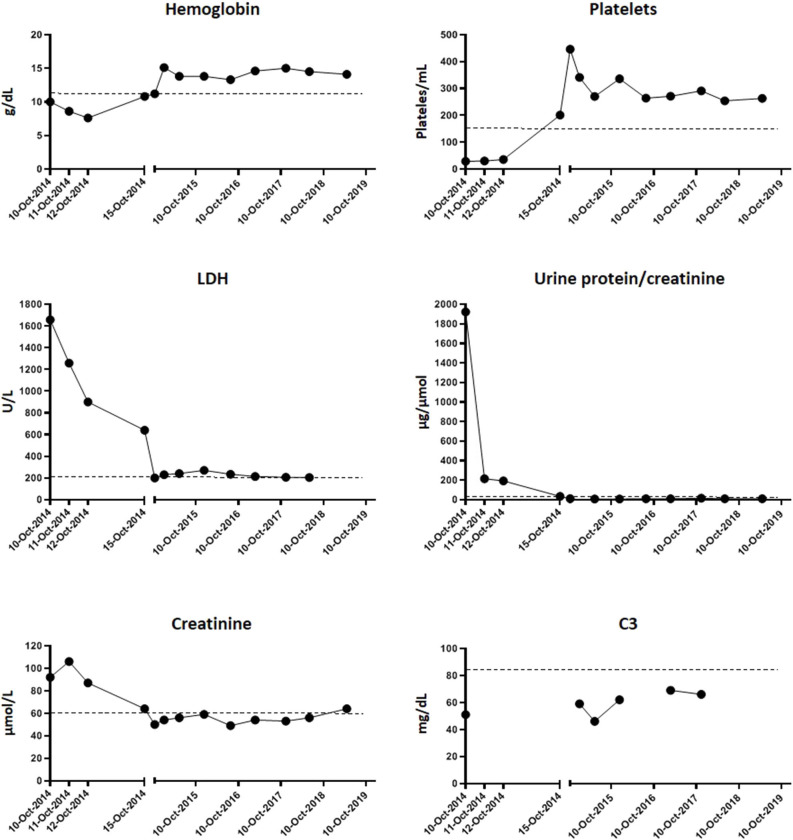
Evolution of hematological parameters and renal activity. Graphics show the blood test determinations since the day of admission (10-Oct-2014) to the day of discharge (15-Oct-2014) and then of the successive routine controls in the following years. Dotted lines represent the upper or minimum value of each parameter from which it is considered pathogenic (hemoglobin > 11.5 g/dl; platelets < 150/ml; LDH > 220 U/L; urine protein/creatinine > 20 μg/μmol; creatinine > 61 μmol/l; C3 < 85 mg/dl).

In October 2014, a previously healthy 8-year-old boy was evaluated at the pediatric emergency room (ER) in a tertiary care hospital for hematuria, asthenia, and mild jaundice observed in previous hours. Nonbloody diarrhea had been present for 3 days. Physical examination was unremarkable apart from mild jaundice. Nonfocal or generalized edema was found. He had no relevant personal or family past history. Initial blood test showed hemoglobin of 12.2 g/dl (>11.5 g/dl), platelets of 35,000/μl (>150,000/μl), creatinine of 84 μmol/L (<61 μmol/L), and normal transaminases, sodium, and potassium. Eight hours later, hemoglobin decreased to 10 g/dl and platelets to 28,900/μl; lactate dehydrogenase (LDH) was determined to be 1,657 U/L (<220 U/L) (Figure 1). Blood test was extended with haptoglobin (undetectable), and a blood smear showed 7–9 schistocytes per field. Creatinine increased to 93 μmol/L. Electrolytes, acid–base balance, and plasma proteins were in normal range. Urine protein to creatinine ratio (UPr/UCr) was 1,921 μg/μmol (<20 μg/μmol). Basic coagulation parameters were normal. Blood pressure remained spontaneously in normal range and diuresis preserved, without involvement of other organs or systems. Patient was admitted to the pediatric ward.

Maximum plasma creatinine was attained on the second day of admission (106 μmol/L). Hemolysis markers started to descend from the third day. He was discharged on the sixth day with hemoglobin of 10.8 g/dl, platelets of 201,000/μl, LDH of 639 U/L, creatinine of 65 μmol/L, and UPr/UCr of 34 μg/μmol (Figure 1).

Additional studies performed during admission revealed the following: plasma homocysteine was normal at 11.4 μmol/L (<15 μmol/L), autoantibodies [antinuclear antibody (ANA), antineutrophil cytoplasmic antibody (ANCA), and antiextractable nuclear antigen (anti-ENA)] were negative, C3 was 51 mg/dl (75–135 mg/dl) and C4 was 21 mg/dl (14–60 mg/dl), and plasma ADAMTS13 activity was 81% (>5%). A stool sample was only obtained after 4 days of admission. Because of its completely normal appearance and the satisfactory evolution of HUS at that moment, it was only tested for Shigatoxin. Blood and urine culture were sterile. The patient was under careful observation during admission without needing renal replacement therapy or any drug. The spontaneous and very favorable evolution, the previous history of diarrhea, and the justification of a negative Shigatoxin assay due to a late stool collection suggested STEC-HUS as the most likely etiology.

**Figure 2:**
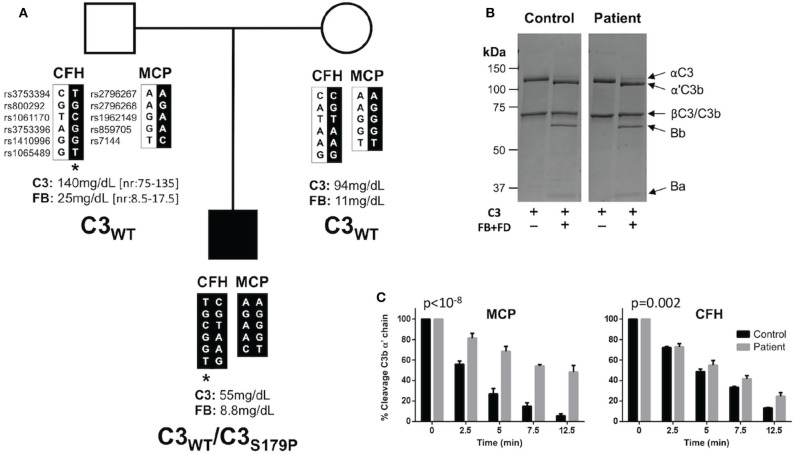
C3_S179P_ is a novel *de novo* gain-of-function mutations that impairs regulation by MCP. **(A)** Pedigree depicting the segregation of *CFH* and *MCP* polymorphisms organized in haplotype blocks. Single nucleotide polymorphisms are identified by their “rs” numbers. In black are the haplotypes inherited by the patient, identified by a solid square. Asterisk identifies the *CFH-H3* aHUS risk haplotype. Plasma levels of C3 and factor B (FB) are indicated for each individual. The patient is the only member of the pedigree carrying the C3_S179P_ mutation. **(B)** C3 purified from the plasma-ethylenediaminetetraacetic acid (EDTA) of the patient activates normally to C3b by factor B (FB) and factor D (FD). Briefly, C3 was purified using a combination of sodium sulfate precipitation, lysine-sepharose chromatography, DEAE-Sepharose anion exchange chromatography, and Mono S HR 5/5 cation exchange chromatography as previously described (15). **(C)** C3b generated from the patient's C3 shows a marked resistant to inactivation by factor I in the presence of MCP. Differences between slopes were tested with a general linear model (GLM), with “time” as an integer variable and “strain” as a nominal one. The time × strain interaction was considered as the estimator of the differences between slopes. MCP (*p* < 10^−8^). FH (*p* = 0.002).

Successive controls showed a complete recovery of renal function and absence of hemolytic activity, anemia, and thrombopenia. No treatment was needed during follow-up. However, decreased C3 levels (46–62 mg/dl) (Figure 1) persisted, and subsequent analysis of factor B plasma levels revealed that they were in the lower part of the normal range (85–170 μg/ml) (Figure 2). Hypocomplementemia is not unusual during a STEC-SHU episode, but the complement normalizes afterwards in these patients (16). The persistent hypocomplementemia in our patients did not correlate with his favorable evolution, raising the suspicion of an underlying constitutive complement abnormality that prompted us to the realization of a complete complement molecular and genetic analysis.

To search for mutations in complement genes, we used an in-house next generation sequencing (NGS) panel including all the complement genes relevant to aHUS (17). A complementary analysis of copy number variations was performed by multiplex ligation-dependent probe amplification (MLPA) with the P236 A1 ARMD mix 1 (MRC-Holland, Amsterdam, Netherlands). These analyses identified a C3 mutation in heterozygosis (c535T>C; p.S179P) that was confirmed by Sanger sequencing. This genetic variant has been found previously associated with aHUS (18). No other genetic alterations were found in this patient. Interestingly, none of his parents present this C3 variation (Figure 2A). Paternity was supported by the analysis of *CFH* and *MCP* polymorphisms. The patient carries the *CFH-H3* aHUS risk polymorphism in heterozygosis, inherited from his father (Figure 2A). He does not carry the *MCPggaac* aHUS risk polymorphism.

Annotation of C3_S179P_ variant with six pathogenicity prediction algorithms (SIFT, POLYPHEN, Mutation Taster, MutAss, FATHMM and CADD) included in the ANNOVAR server (http://annovar.openbioinformatics.org/) indicated that it is most likely a benign C3 variant. However, because the C3 mutations associated with aHUS are gain-of-function mutations that normally are not predicted pathogenic, we purified the C3 protein from the patient's plasma and performed a complete functional characterization following standard procedures in our laboratory (15). These analyses demonstrated that the mutant C3_S179P_ is present in the patient's plasma and shows an altered function with the characteristic of the C3 gain-of-function mutations that associate with aHUS (19–21). Briefly, when purified C3 from the patient was incubated with FB and FD, it completely activated to C3b, suggesting that C3_S179P_ is normally activated by the AP C3 convertase (Figure 2B). When the patient C3b was tested for inactivation by FI in the presence of FH or MCP, we found that it was resistant to inactivation by FI in the presence of both cofactors, but much more resistant when MCP was the cofactor (Figure 2C).

The patient has remained completely asymptomatic without clinical or analytical data of disease activity or renal sequelae for 5 years, with an expectant attitude.

## Discussion

aHUS is a rare, life-threatening renal pathology associated with complement dysregulation. Mutations in genes encoding the regulatory proteins factor H (*CFH*), factor H-related protein 1 (*CFHR1*), MCP (*MCP*), and factor I (*CFI*), as well as mutations in the complement components factor B (*CFB*) and C3 have been found in 50–70% of aHUS patients (13, 22). Importantly, while mutations in the complement regulators are loss-of-function, mutations in complement components like factor B and C3 are gain-of-function (13, 14). For C3, these aHUS-associated gain-of-function mutations result in C3b activated molecules being resistant to regulation by MCP, but not by factor H (19–21). The genetic and functional analyses performed in our patient concluded that he carries a C3 gain-of-function mutation that is prototypical of aHUS. This explains why our patient presents a constitutive complement alternative pathway activation with persistent consumption of C3. He has no familial history of aHUS because C3_S179P_ is a *de novo* mutation, and he is the first in his pedigree carrying this genetic predisposition to aHUS. More interesting is the favorable disease outcome in our patient. Previous studies have shown that C3 mutations, like R161W, tend to be associated with severe aHUS presentations leading to end-stage renal disease. Others, like I1157T, associate with aHUS presentations characterized by multiple recurrences and prolonged favorable outcomes. Interestingly, the presence of the *MCPggaac* aHUS risk polymorphism influences the aHUS presentation in all carriers of C3 mutations (10, 19–21, 23), which may be justified because this polymorphism determines reduced expression of MCP on the cellular surface (8).

Our aHUS registry includes a total of 13 additional patients having a complete clinical record who carry a clearly pathogenic C3 mutation (Table 1). In total, this series comprises five different C3 mutations. Ten of these aHUS patients also carry the *MCPggaac* aHUS risk polymorphism (five in heterozygosis and five in homozygosis) (Table 1). This results in an allele frequency for the MCPggaac polymorphism in this group of patients (*n* = 14) of 0.54, which is significantly different (*p* < 0.0037) from that in the control Spanish population (AF = 0.28; *n* = 107). Notably, the only patient in this series who have had a relative favorable outcome is the only one who does not carry the *MCPggaac* risk polymorphism. Eculizumab treatment was initiated early after aHUS onset or to treat a bad evolution in three patients, and therefore, no conclusions can be reached in them regarding natural progression of aHUS. Notably, nine of the remaining 11 patients reached end-stage renal disease (ESRD) or had multiple recurrences (Table 1). Eight of these patients carry, in addition to the C3 gain-of-function mutation, the *MCPggaac* risk polymorphism or additional pathogenic mutations in the *CFH, CFI, MCP*, and *THBD* genes. The only patient who, like our current patient, does not carry the *MCPggaac* risk polymorphisms or additional pathogenic mutations had a very late onset (63 years old) without recurrences until she was 76 years old. Currently (80 years old), the patient presents chronic kidney disease but does not require hemodialysis. These registry data suggest that the likely explanation for the favorable disease outcome in our patient is that he does not carry additional genetic risk factors, in particular the *MCPggaac* risk polymorphism. A relevant question is why our patient had an aHUS episode. It is known that several viral pathogens interact with MCP and that viral infections may lead to a reduction in the pathogen's receptor. Therefore, one possibility could be that our patient underwent a transient decrease in the cell surface levels of MCP as a consequence of the infection that triggered the aHUS episode. However, this is just a speculation because, when MCP levels were tested, months after the aHUS episode, they were found normal.

**Table 1:** Atypical hemolytic uremic syndrome (aHUS) patients carrying C3 mutations in our aHUS registry.

	**Patient**	**C3 mutation**	***MCPggaac* risk polymorphism**	**Additional changes**	**ESRD**	**Eculizumab**
1	HUS107	Arg161Trp	HET	No	Yes	No
2	HUS316	Lys65Gln	HOM	No	Yes	No
3	HUS416	Lys65Gln	HOM	No	Yes	No
4	HUS500	Lys65Gln	HET	*MCP:* Gly243Val	Yes	No
5	HUS835	Lys65Gln	HET	No	Yes	Yes
6	HUS787	Gln1161Lys	HET	No	No	Yes^a^
7	HUS594	Arg161Trp	HET	No	No	Yes^a^
8	HUS019	Ile1157Thr	HOM	No	No	No
9	HUS612	Lys65Gln	NO	*CFI:* Gly162Asp	Yes	Yes
10	HUS446	Lys65Gln	NO	*CFH:* Arg885Serfs*13	Yes	Yes
11	HUS843	Lys65Gln	NO	No	No^b^	No
12	HUS933	Lys65Gln	HOM	No	Yes	No
13	HUS962	Lys65Gln	HOM	*THBD*: (Ala43Thr)	No	Yes^c^
14	HUS657	Ser179Pro	No	No	No^d^	No

a *Treatment was initiated early after onset; no conclusions can be made regarding the natural progression of aHUS*.

b *Onset at 63 years old without recurrences until she was 78 years old. Treated with five doses of eculizumab she recovered enough renal function to leave hemodialysis. Currently, at 80 years old, she remains with chronic renal insufficiency but does not requires renal replacement therapy*.

c *Very bad evolution of the disease until the administration of eculizumab*.

d *This report*.

Although our patient is currently asymptomatic and presents normal renal function, our functional characterization of the C3_S179P_ variant indicates that it is an important aHUS genetic risk factor. This has important implications. In fact, we cannot exclude that under exposure to a strong environmental trigger (e.g., an infection), our patient will experience a more severe aHUS recurrence. While we strongly recommend normal life to avoid unnecessary anxiety in the patient and its family, we also suggest active surveillance of the patient with specific recommendations. Regular determination of blood pressure, blood count, and measurement of biochemical markers for hemolysis (bilirubin, LDH, haptoglobin), as well as plasma creatinine, proteinuria, and albuminuria are performed. The patient and his parents are instructed to go the ER in case of presenting symptoms suggesting activity of his disease, such as hematuria, oliguria, edema, and significant general malaise in the context of some intercurrent process that can act as a trigger. Ultimately, it is reassuring to know that we have the “magic bullet” of eculizumab, if this patient should need it (24). In conclusion, complete understanding of the etiological factor in the TMA patient is critical to strengthen diagnosis and assist patient management.

## Data Availability Statement

All datasets presented in this study are included in the article/supplementary files.

## Ethics Statement

Written informed consent was obtained from the participant's legal guardian/next of kin for the publication of any potentially identifiable images or data included in this article.

## Author Contributions

JL, MS, and SR designed the study. JL, MS, EA, and SR performed the experiments, collected, and analyzed the data. JL and SR drafted the manuscript, which was revised and approved by all coauthors. All authors contributed to the article and approved the submitted version.

## Conflict of Interest

SR has received honoraria from Alexion Pharmaceuticals for giving lectures and participating in advisory boards. JL has received honoraria from Alexion Pharmaceuticals for giving lectures. None of these activities has had any influence on the results or interpretation in this article. The remaining authors declare that the research was conducted in the absence of any commercial or financial relationships that could be construed as a potential conflict of interest.
